# Development and Psychometric Properties of the Pain and Sensitivity Reactivity Scale in a Diverse Sample of Autistic People

**DOI:** 10.3390/children11121562

**Published:** 2024-12-23

**Authors:** Agustín E. Martínez-González, Matti Cervin, José A. Piqueras, Lidia Infante-Cañete, Susana Pérez-Sánchez

**Affiliations:** 1 Department of Developmental Psychology and Didactics, University of Alicante, Carretera San Vicente del Raspeig, s/n, 03690 San Vicente del Raspeig, Spain; 2 Department of Clinical Sciences Lund, Lund University, 221 00 Lund, Sweden; matti.cervin@med.lu.se; 3 Department of Health Psychology, Miguel Hernández University of Elche, Edificio Altamira, Avda. de la Universidad, s/n, 03202 Elche, Spain; jpiqueras@umh.es; 4 Department of Developmental and Educational Psychology, Faculty of Psychology, University of Malaga, 29010 Malaga, Spain; lidiainfante@uma.es; 5 Hospital Pediatric Service University General “Los Arcos”, 30739 San Javier, Spain; susanaperezsanchez@gmail.com

**Keywords:** autism, pain, sensitivity reactivity, sensory hyporeactivity, sensory hyperreactivity, sensory-over responsivity, sensory-under reactivity

## Abstract

Background: Recent studies indicate the need to examine how the gut microbiota–brain axis is implicated in pain, sensory reactivity and gastro-intestinal symptoms in autism spectrum disorder (ASD), but no scale exists that assesses all these constructs simultaneously. Methods: We created a pool of 100 items based on the real-world experience of autistic people, and a multidisciplinary team and stakeholders reduced this pool to 50 items assessing pain, sensory hypersensitivity, and sensory hyposensitivity. In the present study, we present this new assessment tool, the Pain and Sensitivity Reactivity Scale (PSRS), and examine its psychometric properties in a sample of 270 individuals with autism spectrum disorder (ASD; mean age = 9.44, SD = 4.97), of which almost half (45%) had intellectual disability (ID). Results: A factorial model of three factors (pain, hyporeactivity, and hyperreactivity) and five specific factors for sensory hypo- and hyperreactivity, respectively, fitted the data well. Good to excellent internal consistency and adequate test–retest reliability was found for most PSRS scales. Sound psychometric properties were found in individuals with and without ID. Correlations with other measures of sensory sensitivity and pain indicated sound convergent validity. Conclusions: PSRS shows promise as a reliable measure to analyze pain and sensory reactivity in autistic people regardless of whether they have ID or not. The measure overcomes several limitations of previous assessment tools and includes variables that are important for the understanding of the gut microbiota–brain axis in ASD.

## 1. Introduction

Autism spectrum disorder (ASD) is a neurodevelopmental disorder that is diagnosed based on two main criteria: (1) deficits in social communication and interaction skills, and (2) the presence of restricted and repetitive patterns of behavior [1]. Restricted and repetitive behaviors (RRBs) can be caused by hyper- or hyporesponsiveness to sensory stimuli or unusual interest in sensory aspects of the environment in ASD [1,2].

Altered sensory responsiveness refers to impairments in modulating outputs in relation to sensory stimuli, including in visual, auditive, tactile, smell, taste and proprioceptive modalities [3]. Individuals with sensory difficulties have difficulties regulating and organizing behavioral responses to sensory inputs to match environmental demands. Sensory responsiveness can be classified into three patterns: sensory over-responsivity (SOR: e.g., covering one’s ears in response to sounds), sensory under-responsivity (SUR: e.g., a lack of sensation of loud sounds, slow reaction to pain), and sensation-seeking/sensory interests, repetitions and seeking behaviors (SIRS) [4]. Nonverbal children with ASD are more likely to demonstrate SUR and SIRS compared to peers [5], but few measures of sensory functioning in ASD include SUR and SIRS [3,6]. Research also suggests that SOR, SUR and SIRS commonly co-occur in autistic people, creating significant phenotypic heterogeneity, and both SOR and SUR are included in the diagnostic criteria of ASD [1,3,7,8].

A recent review identified several limitations of current assessment tools of sensory functioning in ASD, with major limitations being (1) that items had not been developed in collaboration with stakeholders, and (2) lack of structural validity analyses (e.g., confirmatory factor analysis) [9]. On the other hand, age, the presence of intellectual disability (ID) and who reports the data (e.g., caregiver versus self-report) are significant moderators in the assessment of SOR [3].

The review also highlighted the difficulty of conducting external validity analyses since the validator measures themselves had unclear psychometric properties. Further, no measure was identified that met the criteria of sufficient psychometric quality according to the COSMIN guidelines [10]. Further, there was a lack of consensus around the terminology (e.g., sensory hyper-reactivity, hyperresponsiveness, SOR) and which components are most relevant to sensory functioning [9]. Last, most current assessment tools do not include specific sensory factors, such as auditory hypersensitivity and auditory hyposensitivity. Thus, there is a need for assessment tools of sensory functioning in ASD that are developed in accordance with leading theories of sensory functioning, in collaboration with stakeholders (e.g., psychologists, pediatricians, educators, families) and where the structural validity of the measure is comprehensively examined.

Uljarević et al. [11] point out that a three-pronged approach should be used when developing measures of sensory reactivity: (1) consider sensory features as dimensional constructs (e.g., tactile hyposensitivity vs. tactile hyperreactivity), (2) examine differences across relevant groups of people (e.g., in people with ASD with and without ID), and (3) move towards comprehensive, multidimensional, and multimodal approaches to the measurement of sensory features, for example, by including pain dimensions.

For the latter, pain is of relevance to sensory functioning in ASD. First, autistic people are at greater risk of experiencing unrecognized pain, especially children with impaired cognitive ability and limited language skills [12]. Second, the frequency and severity of repetitive behavior has a high association with pain [13]. Third, there appears to be a relation between hyposensitivity and pain in ASD, as individuals with ASD show hyposensitivity to subjective pain intensity and affective aspects of pain sensitivity compared to controls [14]. That is, an apparent indifference to pain may be expressed by some autistic people, but this phenomenon has rarely been examined [12], and the relation between sensory reactivity and pain should be analyzed with specific measures [15].

In conclusion, anxiety, SOR, and gastro-intestinal symptoms (abdominal pain and constipation) are possibly interrelated in ASD, and may share underlying mechanisms [16,17]. Further, these variables are all related to the “gut–microbiota–brain axis” [18,19,20,21]. The microbiota–gut–brain axis is a novel field of study where sensory reactions, pain (gastrointestinal symptoms), and gut microbiota can be studied in ASD [18,19].

The present study evaluates the Pain and Sensitivity Reactivity Scale (PSRS), which was developed, in collaboration with stakeholders, to assess sensory hypersensitivity, sensory hyposensitivity, and pain and in autistic individuals with and without ID.

## 2. Materials and Methods

### 2.1. Participants

Caregivers of 270 individuals with ASD (mean age = 9.44, SD = 4.97) participated in this research. The participants were from Spain and came from both urban and rural areas of the following regions: Valencian Community, Murcia region, Madrid, Castilla la Mancha, Castilla y León, Galicia, and Andalusia. Participants with ASD and ID were included when ASD was the primary diagnosis. Table 1 shows the sociodemographic and diagnostic characteristics of the sample.

### 2.2. Measures

#### 2.2.1. Sociodemographic Questionnaire

Lam and Aman’s [22] sociodemographic questionnaire was adapted for the online data collection used in the present study. The questionnaire includes a series of sociodemographic items (e.g., age, sex, country of birth) as well as information on the specific ASD diagnosis and co-occurring diagnoses (e.g., ID).

#### 2.2.2. Social Communication Questionnaire (SCQ)

The SCQ [23,24] is an instrument oriented towards parents or caregivers, with 40 items determining the possible presence of ASD. This instrument has been used for the assessment of ASD in children and adults [25]. It provides a total overall score and three additional scores (Social Interaction Problems, Communication Difficulties, and restricted, Repetitive, and Stereotyped Behaviors). Only the overall score was used in the present study. The present study used Form B of the scale, which assesses behaviors during the past three months. The SCQ has good psychometric properties [24], and showed adequate internal consistency in the present sample (∝ = 0.80).

#### 2.2.3. Repetitive Behaviors Scale-Revised (RBS-R)

The RBS-R is a 43-item instrument oriented to caregivers and mental health professionals which assesses six different dimensions of repetitive behaviors in individuals with ASD and ID: (a) stereotypic, (b) self-injurious, (c) compulsive, (d) ritualistic, (e) sameness, and (f) restrictive [26]. Responses are recorded on a 4-point rating scale ranging from 0 to 3. This scale has been used in both children and adults with ASD [26]. RBS-R has demonstrated adequate psychometric properties for use with individuals with ASD from different countries [27,28]. The internal consistency of this scale in this sample was high (a = 0.95; ω = 0.95).

#### 2.2.4. Short Sensory Profile (SSP)

The SSP [29] is a 38-item caregiver-reported instrument comprised of the items that demonstrated the highest discriminative power of atypical sensory processing based on the Sensory Profile (SP) [30]. Items are scored on a six-point Likert scale. The seven domains of the SSP were based on factor analysis of reported responding in daily life with a normative sample of typically developing children and include tactile sensitivity, taste/smell sensitivity, movement sensitivity, underresponsive/seeks sensation, auditory filtering, low energy/weak, and visual/auditory sensitivity. Studies have shown adequate psychometric properties of the SSP, with internal consistency estimates ranging from 0.70 to 0.90 [31,32,33]. The SSP has been used in children and adults with ASD [33]. In the present study, we used the full scale as well as the tactile sensitivity, taste/smell sensitivity, visual/auditory sensitivity scales, and underresponsive/seeks sensation subscales. The internal consistency of the full scale in the present sample was excellent (∝ = 0.92; ω = 0.91) and all subscales showed adequate internal consistency (∝/ω > 0.70).

#### 2.2.5. Non-Communicating Children’s Pain Checklist—Revised (NCCPC)

McGrath et al. [34] originally developed the NCCPC to measure pain in nonverbal, cognitively impaired children (age range, 3 to 17 years). The NCCPC has been used in children, adults, and patients with varying degrees of cognitive impairment (mean age = 42) [35]. The scale contains 30 items, each rated on a 4-point Likert. Observers are asked to rate the frequency of each item [34]. Studies have found adequate internal consistency in individuals with ASD (Cronbach’s a = 0.72) [36] and ID [37]. The NCCPC-R has shown high internal consistency (Cronbach’s a = 0.93), significant correlations with pain intensity ratings provided by carers, consistency over time, and good sensitivity and specificity in relation to pain [36]. In the present sample, the internal consistency of the NCCPC-R was excellent (∝ = 0.92; ω = 0.92).

#### 2.2.6. Pain and Sensitivity Reactivity Scale (PSRS)

The PSRS is an assessment tool that evaluates reactivity to pain and sensory stimuli using 50 items. The scale is composed of three broad dimensions: pain, sensory hyporeactivity, and sensory hyperreactivity. All items are rated on a 4-point Likert scale ranging from 0 (the behavior does not occur) to 3 (the behavior occurs and is a severe problem). The broader dimensions of hyposensitivity and hypersensitivity include tactile, olfactory, visual, gustatory, and auditory subscales. In addition, the PSRS includes a pain reactivity domain that is assessed using seven items. The PSRS draws in part from the theory of Miller et al. [4] and from neuroscience research showing that pain and sensory reactivity are closely linked [14]. The development of PSRS is described below and its psychometric properties evaluated in the present study.

### 2.3. Procedures

Development of PSRS: The purpose of the development of the PSRS was to create a measure that allows for measuring sensory reactivity and pain in non-autistic and autistic people as well as in individuals with ASD and ID. We adhered to the theoretical model of Miller et al. [4] of SOR and SUR when developing PSRS. Each item is rated on a similar scale to what has been used successfully in other instruments developed for autistic populations (e.g., RBS-R). Items ask for how often a sensation is experienced and to what extent the sensation is a problem. Sensations are indicated as a problem when they are very annoying, very frequent and negatively affect life activities and generate negative consequences for the person or for others.

The PSRS was developed by a multidisciplinary team (3 pediatric specialists, a psychiatrist, two doctoral neuropsychologists, a doctoral psychologist, a neurodevelopmental psychologist, a doctor in chemistry specialized in gut microbiota, a nurse, and two special education teachers). In an initial phase, a pool of 100 SOR, SUR, and pain situations reported by autistic people and their families was generated. Four experts carried out the evaluation (a neuropsychologist with clinical experience in instrument validation; a psychiatrist; a nurse; and a pediatrician). Regarding the pain factor, initially, descriptions of different types of physical pain were included according to the records of the pediatric and psychiatric services. Initially, 32 items related to different physical and medical situations that caused pain were obtained which were then reduced to a shorter item list that included: (1) abdominal pain (e.g., constipation, bloating; 5 items); (2) infectious conditions-related pain (e.g., fever, otalgia; 4 items); (3) skin-related pain (e.g., ulcers, wounds, chafing, eczema, bruises; 5 items); (4) functional pain (e.g., occult fractures, deformities, hangnails, hip/shoulder dislocations, subluxations, spasticity; 3 items); (5) sight and taste-related pain (Eyes: irritation, conjunctivitis, ulcers, wounds; Teeth/Mouth/Throat: caries, canker sores, gingivostomatitis, tonsillitis, abscesses; 4 items). Subsequently, three doctors (two pediatricians and one psychiatrist) screened the items and found that many items were redundant in each of the painful situations, rendering a final set of 7 pain items.

In a second phase, the evaluation of the items was carried out until a total of 50 items were retained. Thus, the examples that were included in each of the 50 items were reviewed and refined. This process was carried out by two special education teachers who are experts in ASD, a pediatrician, a neuropsychologist, and a psychologist.

In a third phase, the clarity of the items was analyzed by three experts. Two were autism experts with experience in instrument development and one expert with more than twenty years of experience in medical assessment. All of them were doctors. The Dunn et al. [38] protocol was applied to all experts to evaluate the items. Judges rated the relevance of each item on a 5-point scale (1: low item clarity; 5: high item clarity), and sought consensus among the experts. Mean item clarity scores were calculated, considering as adequate those items with a score equal to or higher than 3 out of 5 [39]. Items were considered to have an adequate degree of relevance if the V-index was above this cut-off point and the 95% confidence interval did not include the value 0.70 [40]. The mean item clarity scores were above 3.54 in all cases, indicating that the experts considered the items to be clearly worded. In addition, the clinical opinion of the experts was taken into account to indicate the items that were most frequent in individuals with ASD.

Finally, the items were reviewed by caregivers of individuals with ASD. These caregivers had a child with ASD and were aware of situations of sensory reactivity and pain in their children. Their life experience helped improve some examples and clarifications included in the PSRS items. Finally, minor adjustments were made to increase clarity of items.

Recruitment: Causal or incidental sampling was used. Families who had children with ASD in 15 Spanish centers participated. Two centers were specific special education schools; one was residence for people with ASD and ID; eleven were early intervention centers; and one was a regular school with open classrooms. The sample belonged to different sized urban areas, with representation from both rural and urban areas.

A letter with information about the study was sent to a host of stakeholder organizations including education centers, special education centers and associations for families with children with ASD. The center’s director informed the families that they could participate in the study with an online version survey or a paper-and-pencil version. Researchers had an online meeting or a phone call with centers to explain the purpose of the research. Subsequently, the institutions contacted the families to organize a meeting and explain the purpose of the study. Similarly, some institutions facilitated family contacts so that the researchers could directly explain the purpose of the study, and social networks were used to show an explanatory video of the study.

All participating families and caregivers had a child diagnosed with ASD according to DSM-5 criteria [1]. Individuals with ASD with or without ID were diagnosed according to DSM-5 criteria using standardized scales (e.g., Wechsler Nonverbal Scale of Ability, Leiter-3 scale, etc.). The subjects were previously diagnosed by the mental health services and institutions in charge of establishing each country’s degree of disability and dependency. The diagnosis of ASD was made at the early care centers and the pediatric services of the mental health centers of each region. Families with children with another type of neurodevelopmental disorder (e.g., ADHD) were excluded from the study.

The researchers organized a training session for all participating schools in which the purpose of the research study, the instruments used and the administration instructions were described. The tests were administered by experienced psychologists who gave instructions and provided individual assistance to families who needed it. Appropriate instructions were provided for each scale. Participating families completed the protocol at home, and in some cases in a room set up at the center. The researchers could help families resolve doubts about the diagnosis in the first part of the survey, and an explanatory video highlighted the need for the families to consult the psychological and psychiatric reports in case of doubt about the diagnosis. However, the diagnostic part of the protocol was reviewed by the center’s psychologist to detect possible omissions or errors in the severity level of the ASD.

The total time to complete all instruments included in the study was approximately 25 min. After one month, a random sample of 83 caregivers who had a child with ASD completed the study instruments again; this was done to examine test–retest reliability. Participating families did not receive financial compensation for their participation in the study. The study was conducted between June 2020 and May 2022 and was approved by the Ethics Committee of the University of Alicante in Spain (reference: UA-2020-03-27). Caregivers provided informed consent.

### 2.4. Data Analysis

**Figure 1:**
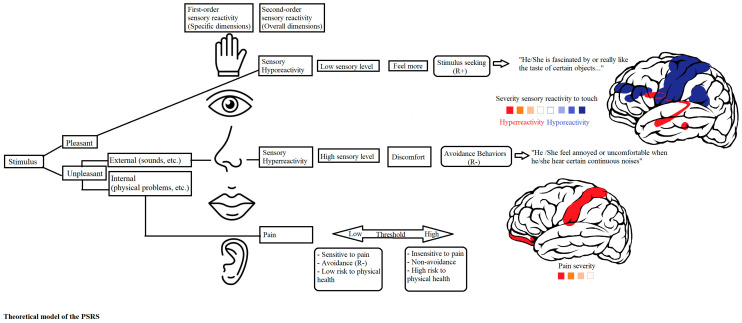
Theoretical model and structure of the PSRS. Copyright © 2023 Martinez-González A.E. [41].

COSMIN Taxonomy of Measurement Properties has been used in the development of the PSRS instrument. First, we evaluated the structural validity of the PSRS (explained above). In short, the PSRS includes a pain scale with 7 indicators/items, a broad sensory hyporeactivity scale with 5 subscales (4–6 items each), and a broad sensory hyperreactivity scale, also with 5 subscales (4–5 items each). In the factor model, we defined the 5 first-order sensory hypo- and hyperreactivity factors to be indicators of the higher, second-order hypo- and hypersensitivity scales, which were then modeled as indicators of a broad third-order sensory reactivity scale (see Figure 1).

To test how well the proposed factor structure explained covariance among responses from the participants, we used the R library lavaan and confirmatory factor analysis (CFA). Because the items of PSRS are ordinal, the diagonally weighted least squares estimator was used. A global evaluation of four fit indices were used to evaluate overall model/data fit: Comparative Fit Index (CFI), Root Mean Square Error of Approximation (RMSEA) and Standardized Mean Square Residual (SRMR). An RMSEA below 0.06, an SRMR below 0.08 and CFI and TLI estimates greater than 0.90 are indicative of acceptable model-data fit; CFI and TLI estimates above 0.95 are indicative of good model-data fit [42]. Scaled fit indices were estimated because of the ordinal nature of the items. Modification indices were evaluated to highlight sources of misfit. The internal consistency of the items of the latent factors were examined by estimating the Cronbach’s alpha (α) and McDonald’s omega (ω) coefficients, and we considered coefficients above 0.70 to indicate adequate internal consistency. The average variance extracted (AVE) was also computed, which estimates the degree of item variance that can be explained by a latent factor. CFA was selected above exploratory factor analysis as the scale had a clear theoretical structure and all items were developed to be used as indicators of pre-defined latent factors.

Construct validity was examined by correlating the empirically derived scores of the measure with the total score of the SSP (convergent validity in relation to the PSRS sensory reactivity scale), the subscale scores of the SSP (convergent and divergent validity in relation to the sensory subscales of PSRS), NCCPC (convergent validity in relation to the PSRS pain scale), RBSR (divergent validity), and SCQ (divergent validity). We expected the corresponding scales (e.g., scales assessing sensory processing) to be more strongly correlated than non-corresponding scale (e.g., scales assessing sensory processing and RRBs). To statistically compare correlations, we used the method presented in Hittner et al. [42], which is implemented in the R library cocor. Test–retest reliability was examined by computing the intraclass correlation coefficient (ICC [2,k]) [43] for the repeated assessments, with values between 0.50 and 0.75 representing moderate reliability, values between 0.75 and 0.90 good reliability, and values greater than 0.90 excellent reliability [10].

## 3. Results

### 3.1. Structural Validity of PSRS

The proposed model explained sample patterns in the data adequately (RMSEA = 0.05, CFI = 0.91, TLI = 0.91, SRMR = 0.10). All item and factor loadings are presented in Table 2. All indicators loaded significantly (*p* < 0.001) onto their proposed first-order latent factor (all standardized loadings > 0.50), and each of the specific hypo- and hypersensitivity factors loaded significantly onto the second-order factors (all standardized loadings > 0.50), which loaded significantly onto the third-order Broad Sensory Reactivity factor. The pain and the broad sensory reactivity factor had a statistically significant positive association/correlation (r = 0.42, *p* < 0.001).

Modification indices suggested that correlated residuals for items 12 and 13 (“Likes touching things and people” and “Hugs people hard”), for the hypo- and hypersensitivity olfactory factors, and for items 9 and 31 (“Scratches wounds until they bleed” and “Feels discomfort by imperfections in the skin”) would improve model/data fit. When adding these parameters, model/data fit was improved (RMSEA = 0.04, CFI = 0.93, TLI = 0.92, SRMR = 0.09) and all additions were statistically significant and showed meaningful parameter estimations (i.e., standardized parameters > 0.30).

### 3.2. Internal Consistency

Internal consistency coefficients for all first- and second-order factors (estimated without the first-order level) are presented in Table 3. All factors showed adequate to good internal consistency except for the hyposensitivity tactile and the hyposensitivity gustatory factors. The internal consistency of the broader factors was good to excellent, but the AVE for the hyposensitivity factor was low.

### 3.3. Convergent and Divergent Validity

The correlations between the two broad PSRS scales (pain and sensory reactivity) and convergent and divergent validator measures are presented at the top panel of Table 4. The correlations between the sensory subscales of the PSRS and the subscales of the SSP are at the bottom panel of Table 4. The PSRS pain scale was significantly and moderately correlated with NCCPC, but this correlation was not significantly stronger than the correlation between the PSRS pain scale and SSP and RBSR. The PSRS pain scale was not significantly correlated with SCQ, and the PSRS pain scale was significantly more strongly correlated with NCCPC than with SCQ.

The PSRS sensory reactivity scale was strongly and significantly correlated with SSP and this correlation was significantly stronger than between the PSRS and all other validator scales except RBS-R. Moreover, moderate to high correlations were found between the sensory hyperreactivity subscales of the PSRS and the hypersensitivity scales of the SSP (between 0.31 and 0.77) and strong correlations between corresponding scales for tactile (r = 0.59), taste (r = 0.77) and auditory (r = 0.60) domains, while moderate correlations emerged for the olfactory (r = 0.41) and visual (r = 0.47) domains. Similarly, moderate to strong correlations were observed in the sensory hyporesponsiveness subscales of the PSRS and the under-responsive/seeks sensation subscale of the SSP (rs between 0.29 and 0.58). The strongest correlations emerged for the tactile (r = 0.58) and auditory (r = 0.53) domains.

### 3.4. PSRS in Participants with and Without Intellectual Disability

We were underpowered to examine measurement invariance (i.e., whether the proposed factor structure was similar) in participants with and without ID. Instead, we examined the internal consistency for the factors in each sample separately. The pain scale showed slightly better coefficients in participants with (α = 0.84, ω = 0.83) versus without (α = 0.72, ω = 0.69) ID. For sensory hyporeactivity, the two groups showed very similar coefficients: with ID (α = 0.85, ω = 0.83); without ID (α = 0.85, ω = 0.85). Similar results emerged for sensory hyperreactivity: with ID (α = 0.90, ω = 0.89); without ID (α = 0.93, ω = 0.93). We also examined whether the two groups differed in their scores using linear regression models accounting for age and sex differences. No significant differences in PSRS factor/scale scores were present.

### 3.5. Test–Retest Reliability

The test–retest coefficients (ICCs) for the pain, sensory hyporeactivity, sensory hyperreactivity, and full sensory reactivity PSRS scales are in Table 5. ICCs were estimated for the full sample and for participants with and without ID, respectively. ICCs for most scales were good to excellent. Of note, the ICCs for the broad sensory reactivity scale as well as the hypo- and hypersensitivity scale were lower for participants with ID.

## 4. Discussion

Sensory reactivity and pain are linked in ASD, and SOR predicts abdominal pain in autistic people [17]. Similarly, children with ASD who have gastro-intestinal symptoms exhibit more irritable and agitated behaviors [44] and significantly higher rates of both anxiety and sensory hyperreactivity [16]. For these reasons, pain and sensory reactivity are important to include in assessment tools of the gut–microbiota–brain relationship in ASD [45].

In the present study, we present a new transdisciplinary and multidimensional instrument that addresses an important gap in the current instrument pool, the PSRS. First, the PSRS includes both sensory sensitivity and pain scales. Second, each scale is derived from leading theories of sensory functioning and developed in collaboration with stakeholders. Overall, our findings support the proposed factor structure of PSRS, with two broad factors (pain and sensory sensitivity), with the sensory factors including a more complex hierarchical structure with separate scales for hypo- and hypersensitivity, which in turn includes subfactors measuring difficulties within each sensory modality (Tactile, Olfactory, Visual, Taste and Audio). Although fit indices were not excellent, small adjustments, accounting for correlations between items with similar content, substantially improved fit, suggesting that the major sources of misfit are not on the structural level. The multilevel structure of PSRS makes it useful in research since it is possible to analyze data on the level that is most beneficial in relation to the research question. The multilevel structure also makes PSRS useful in general practice as it provides specific information about multidimensional sensory and pain difficulties, which is known to be unidentified in many autistic people.

Findings also supported sound internal consistency of most PSRS scales and the internal consistency of the broader factors (sensory hyporeactivity, sensory hyperreactivity and pain) was good to excellent. Lower internal consistency was found for the tactile hyporeactivity factor. These findings are consistent with previous studies that have found lower internal consistency for tactile sensory reactivity scales [46] as well as weak correlations between measure of ASD and tactile sensitivity [47]. However, there is a paucity of studies that analyze the psychometric properties of scales measuring specific sensory dimensions in ASD [32,48]. An explanation of the lower internal consistency of the sensory hyporeactivity scales may be that such difficulties are more difficult for the caregiver to observe and report. In fact, sensory hyporeactivity is less common at younger ages and may be associated with stimulus-seeking behaviors [49,50].

The sensory hypo- and hyperreactivity factors were moderately to strongly correlated, which is in line with our theoeretical framework (see Figure 1) and suggests that alterations in broader underlying mechanisms are involved in the regulation and modulation of sensory processing. The notion of shared underlying mechanisms was further supported by a moderate correlation between the broad sensory sensitivity factor and pain [17].

Convergent validity was supported since the broad PSRS sensory reactivity scale correlated strongly with SSP. However, the moderate correlations in the olfactory and visual dimensions may be due to the fact that the taste-smell and visual-auditory domains are collapsed in the SSP. The broad PSRS sensory reactivity scale also correlated strongly with the RBS-R scale, which assesses RRBs. Although we expected a stronger correlation with SSP than with RBS-R, these results are in line with results showing a clear association between sensory reactivity and RRBs [2]. In fact, sensory reactivity often precedes the onset of repetitive behaviors [1].

We found only a moderate correlation between the pain subscale of the PSRS and the NCCPC, which suggests that the PSRS pain scale assesses aspects that are quite dissimilar to what is measured via NCCPC. Indeed, while the NCCPC only measures the frequency of pain through facial, vocal, and body expressions [34], the PSRS addresses pain through a multidomain measurement framework where frequency, discomfort, and interference are included. Further, the PSRS pain scale is designed for specific pain situations that occur in ASD, and items were developed in collaboration with experienced pediatricians.

A strength of the present study is the inclusion of many individuals with ID and we wanted to examine whether the PSRS was suitable regardless of whether an individual had ID. While we were underpowered to conduct formal invariance tests (e.g., multigroup confirmatory factor analysis), we estimated internal consistency of the broad factors separately in those with and without ID, with internal consistency generally being high in both samples. This is promising and suggests that the items of the broad factors hang together well regardless of ID status. Future studies should include larger samples and conduct formal invariance test. However, it is a challenge to include participants with ID in research since individuals with ID themselves may be unable to report on measures and since families are often burdened. The PSRS includes both self- and observer-reported version, which may help overcome some of these obstacles.

Last, the test–retest reliability, measured approximately one month apart, supported sound psychometric properties of the PSRS. Again, similar results were found in those with and without ID. Test–retest reliability was slightly lower for the sensory hyperreactivity factor in individuals with ID. This may suggest that sensory experiences may vary more over time in this group or that it is harder for observers to adequately report hyperreactivity phenomena in individuals meeting criteria for ID.

The PSRS has a clinical scope in the field of nursing, medicine and psychology because it has an integrative perspective on sensory responses by including sensitivity to pain. Therefore, the pain sensitivity dimension is the greatest contribution of the PSRS. On the other hand, the possibility to obtain different sub-dimensions of SUR with the PSRS (olfactory, gustatory hypo-reactivity, etc.) is another contribution that will help to understand possible subtypes or profiles of sensory reactivity in ASD, and specifically for SUR in the ASD.

The recent literature points to the major limitations of instrument validation studies on sensory reactivity, indicating a lack of research on structural and convergent validity [9]. The present study overcomes some of the limitations of previous studies, including both structural, convergent and discriminate validity. Nevertheless, some limitations merit mention. First, as mentioned above, we were underpowered to conduct formal invariance tests (e.g., invariance by sex, age and ID status). Second, no inter-rater validation of the scale was conducted as all informants were the main caregiver. Third, including a measure of gastro-intestinal symptoms would have further helped validate the PSRS. Fourthly, although attempts have been made to justify that there is a relationship between sensation-seeking and SUR, and that other scales do not include the sensation-seeking variable, it may be that this is an added limitation to the PSRS.

## 5. Conclusions

In conclusion, the PSRS shows promise as a robust measure that can be used to assess sensory reactivity across several domains and pain in individuals with ASD regardless of whether they have ID or not. Future research should expand samples to examine whether the measure works similarly across sexes and age groups. Further, the psychometric properties of the instrument should be analyzed in non-autistic samples. Although the PSRS has fewer items than other similar instruments, a shorter version could be explored, not the least since several items were strongly correlated.

## Figures and Tables

**Table 1:** Sociodemographic characteristics of the ASD sample.

Variables	*n* (%)
**Age**	
4–41 years old	270
**Gender**	
Female	92 (34.1%)
Male	177 (65.6%)
Other	1 (0.4%)
**Country/region of birth**	
Spain	254 (94.1%)
Ecuador	4 (1.5%)
Colombia	1 (0.4%)
Argentina	2 (0.7%)
Chile	1 (0.4%)
Dominican Republic	1 (0.4%)
Other	4 (1.5%)
**Reported diagnosis**	
ASD w/o ID	149 (55.2%)
ASD w Mild ID	37 (13.7%)
ASD w Moderate ID	51 (18.9%)
ASD w Severe ID	32 (11.9%)
**Context**	
Regular class in a regular school	132 (48.9%)
Special class in a regular school	46 (17%)
Special School	52 (19.3%)
Residence	0 (0%)
Residence and Special School	0 (0%)
Day center	0 (0%)
Regular class and Special School	2 (0.7%)
Open classroom	21 (7.8%)
Other	11 (4.1%)

Note. w = with; w/o = without; ASD = Autism Spectrum Disorder; ID = Intellectual Disability.

**Table 2:** Standardized loading of model indicators. All items load statistically significantly (*p* < 0.001) onto the proposed factor.

First-Order	Pain	Hypo Tactile	Hypo Olfactory	Hypo Visual	Hypo Taste	Hypo Audio	Hyper Tactile	Hyper Olfactory	Hyper Visual	Hyper Taste	Hyper Audio
Item 1 [He/she hurts or feels discomfort when he/she has stomach problems]	0.65										
Item 2 [He/she hurts or feels discomfort when he/she has inflammation problems]	0.79										
Item 3 [He/she hurts or feels discomfort when he/she has eye irritation, conjunctivitis, etc.]	0.74										
Item 4 [He/she hurts or feels discomfort when he/she has a fever]	0.63										
Item 5 [He hurts or feels discomfort when he/she has had a fracture or have gone to rehabilitation]	0.52										
Item 6 [He/she hurts or feels discomfort when they prick you for an analysis]	0.70										
Item 7 [He/she hurts or feels discomfort when he/she has fallen or been hit.]	0.54										
Item 8 [He/She prefers very hot or cold water]		0.60									
Item 9 [He/She scratches his wounds until they bleed again.]		0.57									
Item 10 [He/She likes to dress in tight clothes, socks, and shoes.]		0.50									
Item 11 [Squeezes the pen or pencil down a lot when writing.]		0.68									
Item 12 [He/She likes to touch things and people.]		0.64									
Item 13 [Hugs people tight.]		0.59									
Item 14 [He/She is fascinated by certain smells.]			0.94								
Item 15 [He/She smells myself, people and objects.]			0.89								
Item 16 [He/She prefers or likes intense or strong odors.]			0.77								
Item 17 [He/She has a hard time perceiving unpleasant odors or bad odors.]			0.60								
Item 18 [He/She is fascinated by moving or spinning objects.]				0.77							
Item 19 [He/She prefers or likes intense or bright colors.]				0.80							
Item 20 [He/She is attracted to light and reflections.]				0.61							
Item 21 [He/She has a hard time perceiving the strong light before his eyes]				0.59							
Item 22 [He/She is fascinated or really likes the taste of certain objects or parts of the body]					0.77						
Item 23 [He/She likes food with strong flavors.]					0.52						
Item 24 [He/She likes to suck or lick objects, food, etc.]					0.66						
Item 25 [He/She doesn’t feel full/satiated after eating a lot.]					0.66						
Item 26 [He/She is attracted to certain sounds.]						0.64					
Item 27 [He/She listens to TV or music at a very high volume.]						0.60					
Item 28 [He/She likes to make noises or loud sounds.]						0.76					
Item 29 [He/She has a hard time listening to what others are saying, etc.]						0.66					
Item 30 [He/She feels discomfort or discomfort when touched.]							0.64				
Item 31 [He/She feels discomfort or discomfort when he/she notices skin imperfections]							0.69				
Item 32 [He/She feels discomfort or discomfort when touching certain textures.]							0.87				
Item 33 [He/She feels discomfort or discomfort when certain elements or objects come into contact that can touch my head or nails]							0.70				
Item 34 [He/She feels upset or uncomfortable when his/her favorite or usual clothes are not ready.]							0.72				
Item 35 [He/She gets upset or uncomfortable when he/she smells certain odors that other people don’t mind.]								0.91			
Item 36 [He/She feels discomfort or discomfort when he/she smells certain places]								0.95			
Item 37 [He/She feels discomfort or discomfort when he/she smells certain foods.]								0.93			
Item 38 [He/She feels annoyed or uncomfortable when he/she smells certain people.]								0.89			
Item 39 [He/She feels upset or uncomfortable when he/she sees certain colors of food on a plate]									0.87		
Item 40 [He/She feels upset or uncomfortable when he/she sees the physical appearance of some people.]									0.72		
Item 41 [He/she feels upset or uncomfortable when he/she sees a change in something or someone]									0.78		
Item 42 [He/she feels annoyed or uncomfortable when seeing high-intensity light stimuli or bright light.]									0.69		
Item 43 [He/she feels sick or uncomfortable about the taste of certain foods, so he/she only accepts some flavors.]										0.89	
Item 44 [He/she is bothered or uncomfortable by foods with specific textures]										0.79	
Item 45 [He/she feels discomfort or discomfort from foods that are new]										0.86	
Item 46 [He/she feels discomfort or discomfort when changes, even small or subtle, occur in his/her favorite foods]										0.88	
Item 47 [He/she feels annoyed or uncomfortable when he/her hears certain continuous noises]											0.89
Item 48 [He/she feels annoyed or uncomfortable when he/she hears certain sudden, unexpected and intense noises.]											0.89
Item 49 [He/she feels annoyed or uncomfortable when he/she hears loud noises]											0.87
Item 50 [ He/she feels annoyed or uncomfortable when he/she listens to music that is not what he/she usually listens to.]											0.78
Second-Order	Sensory hyporeactivity		Sensory hyperreactivity								
Hypo Tactile	0.68										
Hypo Olfactory	0.59										
Hypo Visual	0.83										
Hypo Taste	0.69										
Hypo Audio	0.84										
Hyper Tactile			0.93								
Hyper Olfactory			0.65								
Hyper Visual			0.83								
Hyper Taste			0.73								
Hyper Audio			0.78								
Third-Order	Broad Sensory Reactivity										
Sensory hyporeactivity	0.83										
Sensory hyperreactivity	0.85										

**Table 3:** Internal consistency of model factors.

	*α*	ω	AVE
Pain	0.83	0.79	44%
Broad Sensory hyporeactivity	0.90	0.89	30%
Hypo Tactile	0.68	0.55	26%
Hypo Olfactory	0.84	0.82	65%
Hypo Visual	0.76	0.73	49%
Hypo Taste	0.69	0.67	43%
Hypo Auditory	0.74	0.70	44%
Broad Sensory hyperreactivity	0.93	0.93	56%
Hyper Tactile	0.85	0.80	53%
Hyper Olfactory	0.95	0.92	84%
Hyper Visual	0.84	0.78	59%
Hyper Taste	0.91	0.88	73%
Hyper Auditory	0.89	0.88	74%

Notes. AVE = Average variance explained.

**Table 4:** Correlations between the broad PSRS scales and validator measures.

	**SSP**	**NCCPC**	**RBS-R**	**SCQ**
PSRS Pain	0.33 **	0.29 **	0.37 **	−0.12
PSRS Broad Sensory Reactivity	0.71 **	0.48 **	0.73 **	−0.16 *
	**SSP Tactile**	**SSP Taste/Smell**	**SSP Under Responsivity**	**SSP Visual/Auditory**
PSRS Hypo Tactile	0.52 **	0.43 **	0.58 **	0.45 **
PSRS Hypo Olfactory	0.34 **	0.25 **	0.29 **	0.30 **
PSRS Hypo Visual	0.48 **	0.40 **	0.49 **	0.44 **
PSRS Hypo Taste	0.39 **	0.34 **	0.48 **	0.37 **
PSRS Hypo Auditory	0.48 **	0.37 **	0.53 **	0.44 **
PSRS Hyper Tactile	0.59 **	0.56 **	0.42 **	0.48 **
PSRS Hyper Olfactory	0.38 **	0.41 **	0.23 **	0.31 **
PSRS Hyper Visual	0.51 **	0.53 **	0.34 **	0.47 **
PSRS Hyper Taste	0.43 **	0.77 **	0.29 **	0.36 **
PSRS Hyper Auditory	0.48 **	0.41 **	0.33 **	0.60 **

Note. * *p* < 0.05. ** *p* < 0.01. SSP = Short Sensory Profile. NCCPC = Non-Communicating Children’s Pain Checklist—Revised. RBS-R = Repetitive Behavior Scale—Revised. SCQ = Social Communication Questionnaire. PSRS = Pain and Sensitivity Reactivity Scale.

**Table 5:** Intraclass correlation coefficients and their 95% confidence intervals for the PSRS scales in the full test–retest sample and in participants with and without intellectual disability, respectively.

	ASD Full Sample *n* = 83	ASD with ID *n* = 23	ASD Without ID *n* = 60
Pain	0.78 (0.73, 0.82)	0.80 (0.72, 0.85)	0.75 (0.68, 0.81)
Broad Sensory Reactivity	0.90 (0.88, 0.92)	0.61 (0.47, 0.71)	0.96 (0.95, 0.97)
Sensory hyporeactivity	0.90 (0.87, 0.91)	0.77 (0.69, 0.83)	0.93 (0.91, 0.95)
Sensory hyperreactivity	0.87 (0.84, 0.89)	0.56 (0.40, 0.67)	0.94 (0.92, 0.95)

Note. ASD = Autism Spectrum Disorder; ID = Intellectual Disability.

## Data Availability

The original contributions presented in this study are included in the article. Further inquiries can be directed to the corresponding author.
